# Commentary: Screens, Teens, and Psychological Well-Being: Evidence From Three Time-Use-Diary Studies

**DOI:** 10.3389/fpsyg.2020.00181

**Published:** 2020-02-18

**Authors:** Jean M. Twenge, Andrew B. Blake, Jonathan Haidt, W. Keith Campbell

**Affiliations:** ^1^Department of Psychology, San Diego State University, San Diego, CA, United States; ^2^Rawls College of Business, Texas Tech University, Lubbock, TX, United States; ^3^Stern School of Business, New York University, New York, NY, United States; ^4^Department of Psychology, University of Georgia, Athens, GA, United States

**Keywords:** digital media, psychological well-being, time use, depressive symptoms, time-diary data

Orben and Przybylski (2019); hereafter, O&P make two primary claims: (a) There is no meaningful link between well-being and screen time measured with time diaries, and (b) the correlations between retrospective and time diary reports of screen time are so low that retrospective reports are not useful.

Regarding the first claim, a simple graph of the time diary data from the primary dataset used by O&P (Figure 1A) shows a clear and substantial relationship between depression and social media, especially for girls, with twice as many heavy (vs. light) users depressed, consistent with other studies (Haidt and Twenge, 2020). Yet O&P made six decisions that substantially reduced the size of the relationship. They chose to:

1. ***Rely solely on linear r***. Linear *r* obscures the curvilinear associations typically found in this area (e.g., the Goldilocks Hypothesis, Przybylski and Weinstein, 2017). For example, the Growing Up in Ireland (GUI) weekend data shows a pronounced curvilinear effect (see Figure 1B). This dataset also shows a substantial association, with nearly twice as many heavy users (12.0%) vs. light users (6.4%) on weekdays having adjustment problems. These effects will appear to be small or null when presented only as linear correlations.
2. ***Include measures of mere participation***. Eighty percent of the variables in O&P's analysis measure mere participation in an activity rather than amount of time spent. Research, including their own, has repeatedly shown that light users (not non-users) of digital media have the highest well-being (Przybylski and Weinstein, 2017). Thus, participation in the activity is not useful for predicting well-being. The relevant issue is heavy use.
3. ***Consider screen time monolithically***. O&P's analysis combines activities more likely to be problematic (e.g., social media) with activities less likely to be problematic (talking on the phone, TV) into a monolithic measure of “screen time.” This mutes practically significant associations with well-being for (e.g.,) social media amid the noise of small or null associations for other activities. Given the wide variety of screen activities (McFarland and Ployhart, 2015), it seems clear that not all screen time is created equal.
4. ***Analyze boys and girls together***. The mental health crisis during the smartphone era has been much more pronounced among girls (e.g., Mercado et al., 2017; Spiller et al., 2019). Thus, it is important to determine if associations between digital media use and well-being are stronger for girls than for boys. That is indeed the case in the time-diary data (see Figure 1A). Without separating the data by gender, the stronger associations for girls are obscured amid the weaker effects for boys.
5. ***Include control variables that are mediators or collider variables***. O&P control for factors including negative attitudes toward school, time spent with parents, parent distress, and closeness to parents that are possible mediators or collider variables. For example, as a collider, parent distress could be caused by the child's heavy screen time or by the child's low well-being. As a mediator, the child's heavy screen time may cause parent distress, which may cause children's low well-being. Best practices guidelines in both psychology and epidemiology state that mediator or collider variables should not be used as controls in correlational analyses: “A solid rule of thumb is that researchers should not control for such posttreatment variables … If mediating variables are controlled for, the very processes of interest are controlled away” (Rohrer, 2018, p. 34, 37; see also Schisterman et al., 2009). In addition, negative attitudes toward school includes well-being items such as “How often do you feel unhappy at school?” In effect, this controls for well-being when well-being is the outcome variable.
6. ***Exclude relevant measures of well-being***. O&P excluded the prosocial behavior subscale of the Strengths and Difficulties Questionnaire (SDQ) with no explanation. Low prosocial behavior was nearly twice as common among heavy gamers (28.7%) vs. light (14.5%). Also without explanation, O&P did not include the Piers-Harris self-esteem items in GUI even though they included self-esteem items from the two other datasets. In GUI, heavy gamers were 73% more likely to have low self-esteem than light users.

**Figure 1:**
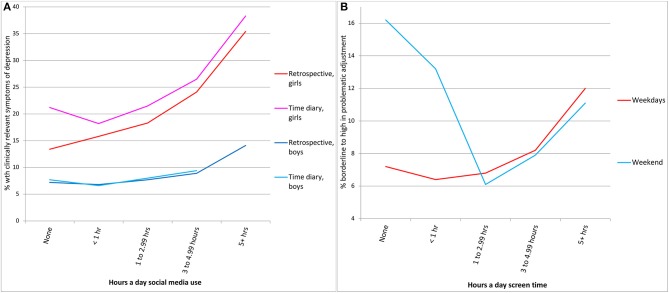
Percent low in well-being by levels of screen time. **(A)** Percent of girls and boys with clinically relevant levels of depressive symptoms by retrospectively reported or time diary hours spent on social media sites, Millennium Cohort Study (MCS). Controlled by age, ethnicity, family income, parent education, parent employment, number of siblings, father present, longstanding illness, and parent word score. Boys reporting 5+h in time diaries were only *n* = 8 and are thus excluded. **(B)** Percent with problematic adjustment on the Strengths and Difficulties Questionnaire by hours per weekday of total screen time in time diaries, Growing Up in Ireland (GUI). Controlled by gender and age.

Turning to the second claim, O&P find low correlations between retrospective report measures and time diary measures of screen time. However, these measures do not ask about the same activities and in one case do not use the same time scale. The GUI retrospective items examine computer use, gaming, and TV/videos, but the time diaries examine internet use, talking on the phone or texting, gaming (with different wording), and TV/videos. Thus, half of the time diary items in the GUI have no direct equivalent in the retrospective items. Even more striking, the retrospective reports in the U.S. data use a scale of “never” to “every day” while the time diaries use minutes per day, also with two different lists of activities.

When similar items are compared, correlations are much higher. In GUI, the retrospective and time diary accounts of gaming on weekdays correlate *r* = 0.33, nearly twice as large as the *r* = 0.18 reported in O&P for all measures combined. This is larger than correlations between self-reports of usual behavior (personality) and multiple-day time diary accounts (largest *r* = 0.25: Rohrer and Lucas, 2018). In fact, the GUI correlation is remarkably strong given that the retrospective accounts ask about a typical weekday while the time diary includes just one weekday, which lowers its predictive value via greater error variance (Iida et al., 2012).

In addition, if retrospective and time diary measures are as different as O&P claim they are, they should show very different associations with well-being. However, that is not the case—the effects are instead very similar (see Figure 1A). Thus, O&P's conclusion that retrospective reports are not useful is not supported by these data.

In conclusion, there is in fact a relationship between adverse mental health outcomes and heavy use (not light use) of some forms of screen-based activity (more than “screen time” in general) for girls (more than for boys), and these relationships appear in both retrospective and time diary reports.

## Author Contributions

JT wrote the first draft of the manuscript and performed analyses. AB obtained and organized the dataset and provided crucial revisions. JH and WC wrote sections of the manuscript and provided crucial revisions.

### Conflict of Interest

JT, JH, and WC have received speaking honoraria from non-profit and for-profit entities for presenting their research, but have not done paid speaking or consulting for any company that would have a particular interest in the links between social media and mental health. The remaining author declares that the research was conducted in the absence of any commercial or financial relationships that could be construed as a potential conflict of interest.
